# Conserved co-expression for candidate disease gene prioritization

**DOI:** 10.1186/1471-2105-9-208

**Published:** 2008-04-23

**Authors:** Martin Oti, Jeroen van Reeuwijk, Martijn A Huynen, Han G Brunner

**Affiliations:** 1 Centre for Molecular and Biomolecular Informatics, Nijmegen Centre for Molecular Life Sciences, Radboud University Nijmegen Medical Centre, Geert Grooteplein 26-28, 6525 GA, Nijmegen, The Netherlands; 2 Department of Human Genetics, Radboud University Nijmegen Medical Centre, Geert Grooteplein 10, 6525 GA, Nijmegen, The Netherlands

## Abstract

**Background:**

Genes that are co-expressed tend to be involved in the same biological process. However, co-expression is not a very reliable predictor of functional links between genes. The evolutionary conservation of co-expression between species can be used to predict protein function more reliably than co-expression in a single species. Here we examine whether co-expression across multiple species is also a better prioritizer of disease genes than is co-expression between human genes alone.

**Results:**

We use co-expression data from yeast (S. cerevisiae), nematode worm (C. elegans), fruit fly (D. melanogaster), mouse and human and find that the use of evolutionary conservation can indeed improve the predictive value of co-expression. The effect that genes causing the same disease have higher co-expression than do other genes from their associated disease loci, is significantly enhanced when co-expression data are combined across evolutionarily distant species. We also find that performance can vary significantly depending on the co-expression datasets used, and just using more data does not necessarily lead to better prioritization. Instead, we find that dataset quality is more important than quantity, and using a consistent microarray platform per species leads to better performance than using more inclusive datasets pooled from various platforms.

**Conclusion:**

We find that evolutionarily conserved gene co-expression prioritizes disease candidate genes better than human gene co-expression alone, and provide the integrated data as a new resource for disease gene prioritization tools.

## Background

In the past few years several bioinformatic tools and approaches have been developed to assist medical genetic researchers in positional candidate disease gene identification (reviewed in [1]; see also [2-5]). Several tools use functional genomics to prioritize candidate genes located within disease-associated genomic loci by evaluating functional relationships between known disease genes and positional candidate genes [6-8]. These tools are based on the premise that genes which are involved in the same disease phenotype are likely to be functionally related [1,9,10]. This has indeed been shown to be the case as evidenced by the fact that these tools all perform better than random expectation in the prediction or prioritization of candidate disease genes. Nevertheless, not all types of functional genomic data perform equally well in terms of sensitivity and specificity [2,7,8]. Microarray expression data have wider coverage than other high-throughput genomic data such as protein-protein interactions, as genome-scale expression analyses are readily and routinely performed with them. Additionally, they are less biased toward better studied genes than gene function annotation or literature mining, although the latter approaches fare better at prioritizing disease candidate genes [2,7,8]. Therefore, given the large coverage of co-expression data and their complementarity to functional annotation and literature mining, it is of importance to maximize the disease gene predictive value of this type of data.

Several bioinformatic candidate disease gene prioritization tools already incorporate microarray-based co-expression data [2,6-8,11,12]. This approach is based on the assumption that if two genes are functionally related then their expression should vary concordantly across tissues and under different circumstances, and proposes that their expression profiles should therefore be correlated. For candidate disease gene prioritization, the use of co-expression analysis is preferable to the use of tissue-specific gene expression patterns, as it is a better predictor of functional relatedness between genes [13].

However, co-expression data can be applied more comprehensively than is currently implemented by these tools. One important and currently underexploited approach is to incorporate co-expression data from other species. One might expect that while human co-expression data are the most relevant for disease gene prioritization, evolutionary conservation of co-expression can be used to enhance the reliability of identified co-expression relationships. The premise is that co-expression relationships that are maintained across phylogenetically distant organisms must be under selective pressure, and should therefore be functional – a premise that has indeed been confirmed in several previous studies [14-17]. Though one tool already includes multi-species co-expression data [11], the improvement in disease gene ranking performance due to the exploitation of evolutionary conservation has not yet been investigated.

We therefore investigated the predictive value of conserved co-expression for candidate disease gene prioritization. To this end we analyzed how well co-expression between known and candidate disease genes could prioritize positional candidate disease genes. We restricted our analysis to known disease genes from genetic diseases containing at least two known causative genes. We constructed artificial loci of 100 candidate genes around the known disease-causing genes, and investigated the tendency of these causative genes to have higher co-expression with other known causative genes compared to the non-causative candidate genes from the same disease loci. Using co-expression data from five eukaryotic species – baker's yeast (*Saccharomyces cerevisiae*), nematode worm (*Caenorhabditis elegans*), fruit fly (*Drosophila melanogaster*), mouse (*Mus musculus*) and human – we investigated the effect of evolutionary conservation on the ranking of the disease gene pairs, finding that evolutionary conservation of co-expression does indeed improve disease gene ranking. Therefore, exploiting evolutionary conservation could potentially improve the performance of co-expression data in existing disease candidate gene prioritization tools [2,6-8], which might in turn improve the prioritization of less well-studied genes.

## Results

### Evolutionary conservation of co-expression improves disease gene ranking performance

We investigated how well disease genes tend to rank relative to non-causative candidate disease genes when ranked according to co-expression with other genes known to cause the same disease. We combined co-expression scores across species using orthology relationships from the euKaryotic clusters of Orthologous Groups (KOG) database [18]. The co-expression scores are thus based on these KOGs rather than on individual genes (see methods section for further details). We used expression data from human, mouse, fruit fly (*D. melanogaster*), worm (*C. elegans*) and baker's yeast (*S. cerevisiae*) assembled from the Gene Expression Omnibus database [19] and the Genomics Institute of the Novartis Foundation [20] Gene Atlas expression data. For this study we used artificial disease loci containing 100 genes per locus. Testing with 50, 100 and 200 genes per locus does not make much difference though smaller loci tend to perform slightly better than larger loci (data not shown). Only disease gene pairs with co-expression scores unlikely to occur randomly in the corresponding dataset (i.e. more than 2 standard deviations from the dataset randomization mean) were included in the final rankings. This process implicitly weighs the scores according to the number of species involved, as the random score distributions are narrower for datasets combining more species. The standard deviations of these randomized distributions range from 0.051 for the five-species combined dataset to between 0.057 (yeast) and 0.094 (mouse) for the individual single-species datasets. Therefore, a given correlation score is more likely to be considered significant in a multi-species dataset than in a single-species dataset.

**Figure 1:**
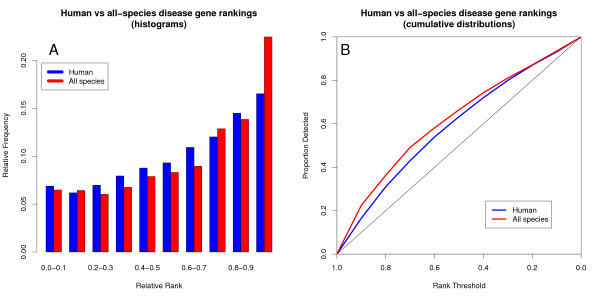
**Evolutionary conservation of co-expression improves disease candidate gene prioritization performance over human-only co-expression**. The disease gene rank histogram shows the relative proportions of disease genes scoring in different rank bins based on co-expression with known disease genes causing the same disease. Disease gene rank indicates its degree of co-expression with known disease genes, relative to those of the other locus candidate genes. Evolutionary conservation results in a much higher proportion of disease genes ranking in the top 10% of the locus candidate genes and fewer in the mid-regions of the lists. A) Disease gene rank histogram. B) Cumulative proportion of disease genes detected as the co-expression rank threshold is decreased from 1 to 0.

Genes whose co-expression is conserved through evolution have been shown to have stronger functional ties than genes whose co-expression is not [17]. Therefore, conserved co-expression between species should improve disease gene ranking over co-expression in one species alone. Such a trend is indeed apparent (Figure 1). Both for human and for multi-species KOG-based co-expression sets, disease gene pairs generally score in the upper half of the co-expression rankings for the KOG-mappable genes in the disease loci and are significantly better than random expectation (medians 0.64 and 0.69 for human-only and conserved co-expression sets respectively; p << 10^-16 ^for both sets, Wilcoxon signed rank test). Therefore, the causative disease gene in a candidate locus will generally rank higher than the other locus candidate genes. Furthermore, the multi-species combined co-expression set performs significantly better than the human-only co-expression set (medians 0.64 and 0.69 respectively, p < 10^-6^, Wilcoxon rank sum test), indicating that the use of evolutionary conservation can significantly improve co-expression-based candidate disease gene ranking.

**Figure 2:**
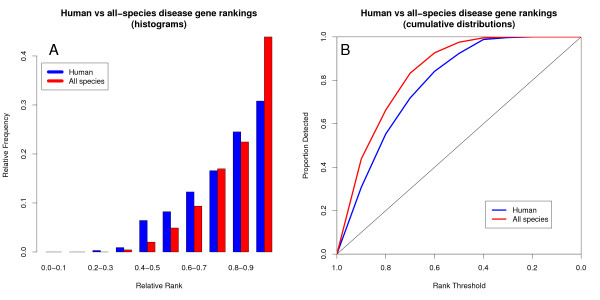
**Filtering out insignificant co-expression improves performance**. When disease gene pairs with co-expression scores that do not differ significantly from those of randomized datasets are filtered out, the disease gene ranking performance improves substantially. However, the coverage drops from 3286 to 695 pairs. A) Disease gene rank histogram. B) Cumulative proportion of disease genes detected as the co-expression rank threshold is decreased from 1 to 0.

Given that co-expression-based disease gene ranking is only sensible if there is a non-random co-expression relationship between the disease genes, we further narrowed down the analysis to only those disease gene pairs that are likely to be genuinely co-expressed, having scores that are unlikely to occur randomly in their datasets. This substantially improves the rankings (Figure 2), at the expense of reduced coverage (695 versus 3286 disease gene rankings). Both the human-only and the conserved co-expression datasets now rank the disease genes very high (medians of 0.87 and 0.93 respectively), with the conserved co-expression still significantly outperforming the human-only co-expresssion (p < 10^-11^, Wilcoxon rank sum test). A much higher proportion of disease genes is now ranked in the top 10% of the candidate gene lists, and the use of evolutionary conservation increases this proportion by almost half, from 31% to 44% of the ranked disease genes.

### Disease gene ranking improved by co-expression conservation at different evolutionary distances

**Figure 3:**
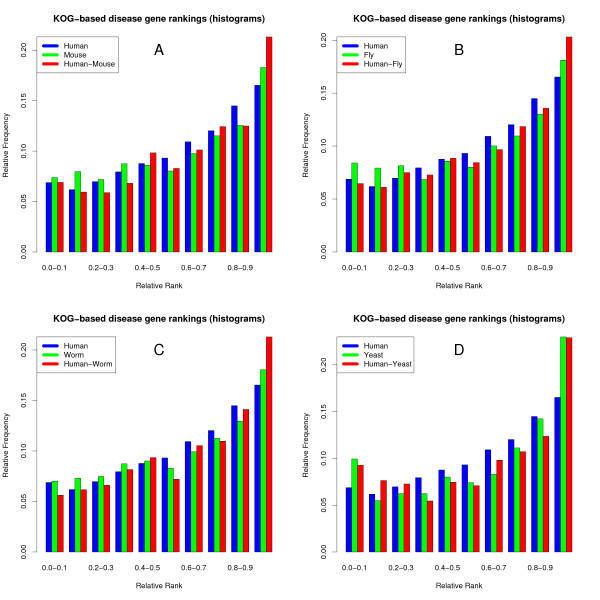
**Co-expression-based disease gene rankings are improved by conservation at various evolutionary distances**. Pairwise cross-species co-expression improves disease gene ranking over single-species datasets for comparisons between human and mouse (A), fruit fly (B), and nematode worm (C) co-expression. However, co-expression in yeast alone performs as well as co-expression conserved between human and yeast (D), and significantly better than human-only co-expression (p = 0.01).

Evolutionary conservation across multiple species clearly improves the disease gene ranking performance of gene co-expression. However, what influence might different evolutionary distances have on this improvement? Is the evolutionary distance between human and mouse sufficient to improve co-expression performance, and is yeast biology so divergent that it would reduce rather than improve performance? To examine the role of evolutionary distance on the disease gene ranking performance of gene co-expression, we conducted pairwise comparisons in which we compared the ranking performance human co-expression with co-expression conserved between human and each of the other species. For all pairwise comparisons except the human-yeast comparison, evolutionary conservation significantly improves co-expression-based disease gene ranking compared to using only data for human genes (Figure 3, Table 1). Surprisingly, even human-mouse conservation improves disease gene ranking despite the relatively short evolutionary distance between these two species.

**Table 1:** Pairwise species comparisons for co-expression-based disease gene ranking.

Individual species	# Disease gene pairs	Median rank	Pairwise combined sets	# Disease gene pairs	Median rank	P-value (Combined better than single species)
Human	3286	0.64	Human-Mouse	3114	0.67	6.7 × 10^-4 ^*
Mouse	3188	0.63				1.3 × 10^-5 ^*
Human	3286	0.64	Human-Fly	3176	0.66	0.007 *
Fly	3534	0.62				1.8 × 10^-5 ^*
Human	3286	0.64	Human-Worm	2954	0.67	2.1 × 10^-4 ^*
Worm	3264	0.63				9.1 × 10^-6 ^*
Human	3286	0.64	Human-Yeast	550	0.67	0.12
Yeast	674	0.69				0.74

* statistically significant at p = 0.05 level

In contrast to the other species, co-expression conservation with yeast does not significantly improve disease gene ranking (Table 1). For this species pair yeast-only co-expression performs best, outperforming even the combined human-yeast set at ranking human disease genes (albeit not significantly; p = 0.26). This is primarily due to specific disease types involving housekeeping processes such as metabolism (congenital disorder of glycosylation, glycogen storage disease) and DNA repair (xeroderma pigmentosum) which consistently score well particularly in the yeast set. As yeast co-expression already performs very well, the combination with human co-expression may not yield much extra information. However, this performance comes at the expense of much reduced coverage of disease genes relative to the other sets, which all have a similar coverage (550 disease gene rankings for the human-yeast set, versus ~3000 for human-mouse, human-fly and human-worm sets). It is thus evident that despite the large evolutionary distance between these two species, yeast co-expression is still effective at ranking human disease genes for those genes that have orthologs in both species.

### Disease gene ranking performance is dependent on co-expression data used

**Figure 4:**
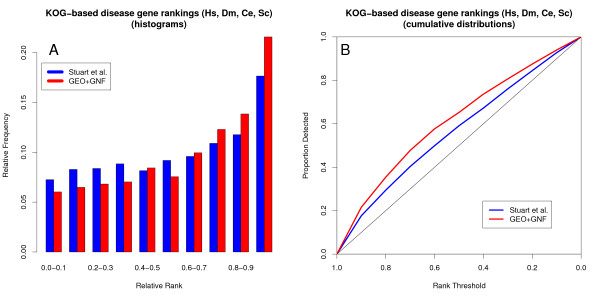
**Single platform co-expression dataset outperforms multiple platform co-expression dataset**. The more cohesive GEO/GNF set containing a single microarray platform per species outperforms the more inclusive multi-platform Stuart et al. set (p < 10^-10^). Both sets have similar coverage, with 3212 and 3286 disease gene pairs for the Stuart et al. and GEO/GNF sets respectively. These results are based on co-expression data from human, fly, worm and yeast, with co-expression scores are averaged across species wherever possible. A) Disease gene rank histogram. B) Cumulative proportion of disease genes detected as the co-expression rank threshold is decreased from 1 to 0.

We initially used the multi-species co-expression dataset from Stuart and colleagues [21], but this resulted in limited disease gene ranking performance. We suspected that the extensive pooling of expression data from different platforms might have a negative impact on performance. Therefore, we created our own custom multi-species co-expression dataset, in which we restricted ourselves to a single microarray platform per species. Consistent with the findings of others [22,23], this single platform approach resulted in significantly better performance, even when using only the four species included in the Stuart et al. dataset (Figure 4). Though both datasets tend to rank disease genes highly, the new (GEO/GNF) dataset performs significantly better than the larger and more inclusive dataset from Stuart and colleagues (p < 10^-10^, Wilcoxon rank sum test) without loss of coverage (3286 and 3212 disease gene rankings for the GEO/GNF and the Stuart et al. datasets respectively).

In addition to restricting expression data to a single platform per species, normalizing the microarray expression data according to total expression level also improves the ranking of disease genes relative to non-disease genes from the candidate loci. As we were mainly interested in relative expression levels of genes across conditions and not in total gene expression levels, we normalized all expression values according to the total expression level of the microarray sample (see methods section for further details). This reduces systematic biases between samples due to differences in total expression levels and highlights the expression relationships between genes per sample, resulting in up to 5% improvement in candidate disease gene ranking (data not shown).

All disease gene ranking and conserved co-expression correlation data presented here are freely available online [24].

## Discussion

In this study, we show that we can increase the predictive value of co-expression for disease gene prioritization by exploiting evolutionary conservation, despite the variations in the biology of the species compared. Given a genuine co-expression relationship between the disease genes, using conserved co-expression to prioritize candidate disease genes can reduce the number of genes to be tested over sevenfold compared to using a random ranking of the candidate disease genes, as the correct gene will be found on average after testing 7% of the candidates (the median disease gene rank is 0.93) instead of 50% (Figure 2). Encouragingly, even human-mouse conservation can lead to a substantial improvement in disease gene ranking performance, despite the relatively short evolutionary distance between these two species (Figure 3). This means that improvements in specificity can be gained without large losses in sensitivity, as most human genes have mouse orthologs.

An interesting finding is that large pooled datasets which combine as many expression data as possible from various experiments and platforms can actually result in reduced co-expression performance relative to smaller but more coherent expression datasets (Figure 4). Microarray data are notoriously variable between independently generated datasets while they are somewhat more consistent between experiments using the same platform [22,23]. In order to minimize dilution of the co-expression signal when combining data from many different sources a weighting scheme is required, such as using the co-expression overlap between different sets to weigh the relevance of the co-expression value [25]. Our results are consistent with these previously reported findings, as our single-platform-per-species dataset ranks disease genes significantly better than the more inclusive pooling approach adopted earlier by Stuart and colleagues [21]. An alternative explanation would be that their expression sets are of lower quality or are less representative of the relationships between disease genes, but there is no reason to assume that either of these is the case. This underscores the fact that combining as many data as possible does not necessarily lead to an improved performance of co-expression data for disease gene prioritization, so it is therefore not a trivial finding that combining data from different species does.

Another reason why the larger sets do not perform as well as the smaller sets could lie in the use of correlation coefficients to determine genetic relatedness. Correlation coefficients estimate expression coherence across all conditions surveyed, but even functionally related genes may not have coherent expression patterns across all tissues and conditions. The larger the datasets, the greater the potential for irrelevant conditions to mask the co-expression relationship that a group of genes has under a limited set of conditions. Therefore, a biclustering-based approach [26,27] may yield more refined co-expression relationships between genes, and is a potential avenue for future improvement of co-expression-based disease candidate gene prioritization.

## Conclusion

We analyze here the predictive power of gene co-expression for disease gene prioritization and identify factors that affect it, such as evolutionary conservation. We show that co-expression data from other species have predictive power for human disease gene prioritization, and that evolutionarily conserved gene co-expression can improve disease gene prioritization over human-only gene co-expression. In addition, we show that platform consistency is important and that smaller but more cohesive datasets can outperform larger pooled datasets. Though we only examined disease gene ranking, these findings have broader relevance for the use of microarray co-expression data in functional genomics. We provide these conserved co-expression data as a new resource that can be used in disease gene prioritization programs, particularly those that integrate several different data types.

## Methods

### Disease data

We used the Online Mendelian Inheritance in Man (OMIM) [28] Morbid Map as a source of genetic diseases and known disease genes. We restricted our analysis to those diseases with two or more known disease genes. There were 890 known disease genes (727 distinct genes) for 177 diseases in our dataset. Artificial disease loci were constructed around these known disease genes based on localization information from the Ensembl database [29], by taking the required number of neighboring genes centered on the disease gene. These genes were then translated to HGNC gene IDs [30] or KOG IDs [18] depending on the analysis. This means that the locus genes used in the analyses are a subset of the Ensembl genes in the locus, depending on how many could be mapped to their relevant IDs. We used artificial loci of 100 genes, which is representative for the average candidate disease locus, as the OMIM heterogeneous disease loci have a median of 88 genes per locus. In addition, we investigated the use of 50- and 200-gene artificial loci, as well as actual associated loci from OMIM Morbid Map, but do not consider them further as their results did not differ substantially from those of the 100-gene artificial loci.

### Expression data processing

Initially we used the multi-species expression data used by Stuart and colleagues in their functional analysis of conserved co-expression [21]. This dataset contains expression data for human, fruit fly (*Drosophila melanogaster*), nematode worm (*Caenorhabditis elegans*) and baker's yeast (*Saccharomyces cerevisiae*) genes. These expression data had already been normalized and were therefore not further processed prior to their use in the co-expression calculations.

However, due to limited performance of this dataset in ranking disease genes we created a new multi-species co-expression dataset involving expression data from these four species and mouse (*Mus musculus*). For human and mouse expression data we used the gcRMA-normalized Gene Atlas expression sets generated by the Genomics Institute of the Novartis Foundation [20], as this is an often-used and well-constructed expression dataset. The expression values were log_2_-transformed to increase robustness and emphasize the variation between the lower expressed genes relative to the more highly expressed ones. Lacking similar standard expression datasets for the other species, the expression data for fruit fly (*Drosophila melanogaster*), nematode worm (*Caenorhabditis elegans*) and baker's yeast (*Saccharomyces cerevisiae*) were collected from the Gene Expression Omnibus (GEO) database [19] of the National Center for Biotechnology Information (NCBI). In order to maximize consistency of the expression data, only one expression platform was used per species. As these expression data are normalized in different ways in different experiments, we used the raw signal intensity data (Affymetrix CEL files) rather than the normalized expression data. This further restricted the data available to us, as these raw data are not required for submission of expression data to the GEO database and are not included with all datasets. Only experiments with at least 10 microarray samples were considered. We ended up with 357 samples in 12 experiments for yeast, 242 samples in 8 experiments for fly and 123 samples in a single experiment for worm (Table 2). For within-array normalization we used the Robust Multi-array Averaging (RMA) algorithm [31] as implemented in the R statistical software bioconductor library [32]. However, our between-array normalization was done by normalizing for total sample expression (see below), as we found this to yield better results than RMA-normalizing per experiment (data not shown). Total expression normalization prevents spurious correlation due to differences in total expression level between samples. For the yeast and fly datasets, data were pooled across all experiments before total expression normalization and calculation of the co-expression correlation coefficients.

**Table 2:** GEO expression datasets used

Species	GEO series ID	Number of samples	Reference
Fly	GSE6515	78	[36]
	GSE1690	10	[37]
	GSE2780	10	[38]
	GSE2828	12	[39]
	GSE3069	18	[40]
	GSE3842	72	[41]
	GSE4714	30	[42]
	GSE4235	12	[43]
Worm	GSE2180	123	[44]
Yeast	GSE4807	30	[45]
	GSE6073	12	[46]
	GSE1311, GSE1312, GSE1313	66	[47]
	GSE1639	18	[48]
	GSE1693	26	[49]
	GSE1934	24	[50]
	GSE1938	15	[51]
	GSE1975	28	[52]
	GSE2343	12	[53]
	GSE3076	96	[54]
	GSE3821	16	[55]
	GSE4135	14	[56]

The Affymetrix probe set expression levels were translated to gene expression levels, and for genes that were represented by multiple probe sets the median was taken. These genes were further filtered according to gene sets used (Table 3). Both GEO and GNF datasets were further normalized according to total sample expression level by dividing each gene expression value by the mean expression value of all considered genes in the microarray sample. This further minimizes spurious correlations due to differences in total expression level between experimental conditions or across tissues.

**Table 3:** Genes included in the co-expression calculations

Species	Genes included in co-expression data	Number of genes
Human	Those with gene names also present in Affymetrix HG-U133A microarray platform	13955 (11410 genes in all disease loci combined)
Mouse	Those with mouse gene names (unknown transcripts, RIKEN transcripts and predicted genes excluded)	14000
Fly	Those with FlyBase IDs	13282
Worm	WormBase-annotated genes	17948
Yeast	Those with systematic names (Y...IDs)	6563

No artificial cut-off was used to filter out noisy low expression values, or to define presence or absence of gene expression in a sample. This is not necessary, as we are using correlation between expression profiles rather than absolute expression levels. The inclusion of non-biologically significant noise should not result in spurious correlations between genes, and if there is a correlation between low expression values then they are probably not merely noise and should not be filtered out.

It should be noted that while we used log_2_-transformed signal intensity values in a multi-species study involving different microarray platforms, and not relative abundances as Liao & Zhang did [16], our analysis does not suffer from the same problems that led them to use relative abundances. We do not directly compare expression values between different microarray platforms. Instead, these expression values are converted to co-expression values for each platform separately. This process involves only within-platform signal intensity comparisons. The between-species – and therefore between-platform – comparisons are done at the co-expression level and involve comparisons of Spearman rank correlation coefficients.

### Co-expression score calculations

We used Spearman rank correlation coefficients as the microarray signal intensity values were not normally distributed. For the GEO datasets comprising several experiments (the fly and yeast sets), these data were pooled before gene pair co-expression correlation coefficients were calculated.

In order to be able to compare co-expression relationships between species, we used the gene orthology relationships as defined by the euKaryotic clusters of Orthologous Groups (KOG) database [18]. We chose to use KOGs instead of a metagenes-based approach such as was used by Stuart and colleagues [21] in order to maximize coverage, as KOGs not only contain bidirectional best hits but also closely related paralogs. The gene to KOG mapping was done using the STRING database version 6.1 [33]. Mapping of the protein IDs used in STRING to the gene IDs used on the microarrays was done using Ensembl BioMart [34]. Of the 13955 human genes with expression data used in this study 8186 could be mapped to KOGs.

**Figure 5:**
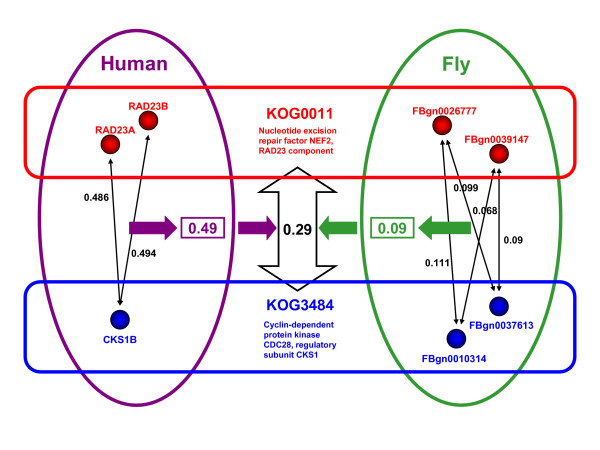
**Procedure for calculating conserved co-expression scores**. The procedure is illustrated using an example involving KOGs KOG0011 and KOG3438 between human and fly. KOG0011 contains two genes in each species (RAD23A, RAD23B and FBgn0026777, FBgn0039147 in human and fly respectively) while KOG3438 contains one in human (CKS1B) and two in fly (FBgn0010314 and FBgn0037613). For each species a KOG0011-KOG3438 co-expression (thick purple and green arrows) correlation is calculated by taking the mean of all gene-gene combinations (thin black arrows) for the two KOGs. The mean of these species-specific KOG-pair correlations (thick vertical orange arrow) is taken to represent the final multispecies KOG0011-KOG3438 co-expression correlation. This co-expression value is used for all relevant gene-pairs as their KOG-based co-expression score. If co-expression is conserved in both species then this value will be high, if it is high in only one species it will be intermediate, and if it is low in both it will be low.

A single pair of KOGs can have multiple co-expression values if one or both of them contain multiple genes per species. In such a case these co-expression scores need to be combined into a single co-expression score representative of all pairwise combinations of genes in the two KOGs (Figure 5). We accomplished this by taking the mean of all such gene pair co-expression scores, resulting in a single KOG-based co-expression score (within-species averaging).

In order to incorporate evolutionary conservation into the final co-expression scores, we took the mean of the species-specific KOG-based co-expression scores over all species considered (between-species averaging). For the comparison between human and multi-species conserved co-expression the union of all the sets was taken for maximal coverage – i.e. all KOG-based co-expression scores were used regardless of which species were represented in the KOGs.

### Disease gene ranking analyses

**Figure 6:**
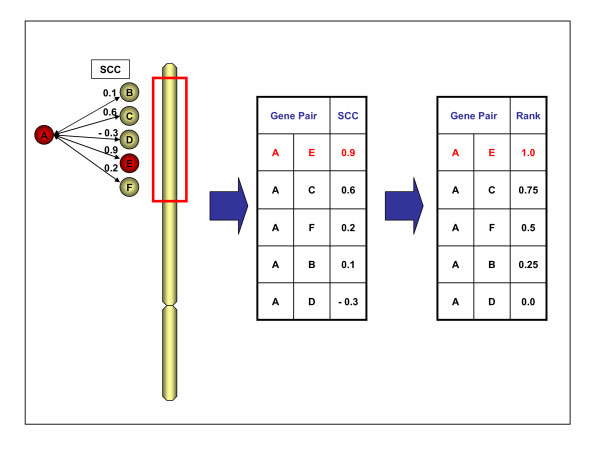
**Disease gene ranking procedure**. Co-expression Spearman correlation coefficients (SCC) are determined for all candidate disease genes from a disease locus with known causative gene (E) and another gene known to cause the same disease (A). The SCCs are ranked, and the ranks are subsequently normalized to the 0.0–1.0 range. The relative position of the causative gene in the locus (E) is determined. This procedure would subsequently be repeated with E as known disease gene and A as candidate disease gene. For each disease, each gene is treated as a candidate disease gene in turn and its co-expression is successively compared to all other genes known to cause the same disease, leading to a list of scores per disease.

We investigated the co-expression ranking performance between candidate disease genes and known disease genes for each pair of disease genes causing the same disease (Figure 6). To this end we ordered all co-expression values between a known disease gene and the genes in a candidate disease locus, and scored the relative rank (0–1) of the actual causative gene in the resultant list. If the causative gene was at the top of the list it was assigned a relative rank of 1.0, and if at the bottom it received a relative rank of 0.0. A score of 0.5 indicates an equal number of more highly co-expressed and less highly co-expressed non-disease genes in the candidate locus, and is equivalent to random expectation. For each disease each causative gene was sequentially treated as the known disease gene and tested against all the other loci.

To avoid ranking candidate disease genes which do not have any co-expression relationship with each other at all, we randomly permuted the co-expression datasets used to determine the random distribution of co-expression scores for each dataset. We then excluded all disease gene pairs for which the co-expression score fell within 2 standard deviations of the randomization means. These distributions all had co-expression scores with a mean of approximately zero and standard deviations ranging between 0.05 and 0.09 depending on the dataset. Multiple randomizations always resulted in almost identical score distributions per dataset due to the large numbers involved.

To investigate the influence of evolutionary conservation on disease gene pair ranking performance, human-derived co-expression data were compared with co-expression data averaged across all five species included in the study. Additionally, pairwise species comparisons were performed comparing human-only co-expression data with pairwise conserved co-expression between human and mouse, fly, worm or yeast.

In order to test for the effect of averaging gene-gene co-expression within KOGs, the performance of the GNF human expression set when using KOG-based co-expression was compared to its performance when using gene-based co-expression.

### Tools

The R statistical software package [35] was used for the microarray data processing and the Spearman rank correlation calculations, as well as for statistical tests and data plotting. For performance reasons, small custom-written C++ programs were used to average the gene-gene correlation coefficients into KOG-KOG correlation coefficients, and the per-species KOG-KOG correlations into cross-species KOG-KOG correlation values. Python scripts were written for the disease gene correlation coefficient ranking analyses. All scripts and source code are available on request.

## Authors' contributions

MO and JvR carried out the analyses. MO drafted the manuscript. MAH and HGB conceived of the study and supervised the analyses and manuscript drafting. All authors participated in the design of the study, and read and approved the final manuscript.
